# Genome-wide analysis of the *Tritipyrum* WRKY gene family and the response of *TtWRKY256* in salt-tolerance

**DOI:** 10.3389/fpls.2022.1042078

**Published:** 2022-12-14

**Authors:** Kuiyin Li, Xiaojuan Liu, Fang He, Songshu Chen, Guangyi Zhou, Yuhai Wang, Luhua Li, Suqin Zhang, Mingjian Ren, Yuanyuan Yuan

**Affiliations:** ^1^ Guizhou Subcenter of National Wheat Improvement Center, College of Agronomy, Guizhou University, Guiyang, China; ^2^ Anshun University, Anshun, China; ^3^ Zaozhuang University, Zaozhuang, China; ^4^ Jinan Academy of Agricultural Sciences, Jinan, China; ^5^ Yantai Academy of Agricultural Sciences, Yantai, China

**Keywords:** *Tritipyrum*, WRKY, salt-tolerance, *TtWRKY256*, genome-wide, expression patterns

## Abstract

**Introduction:**

The transcription factor WRKY is widespread in the plant kingdom and plays a crucial role in diverse abiotic stress responses in plant species. Tritipyrum, an octoploid derived from an intergeneric cross between Triticum aestivum (AABBDD) and Thinopyrum elongatum (EE), is a valuable germplasm resource for introducing superior traits of Th. elongatum into T. aestivum. The recent release of the complete genome sequences of T. aestivum and Th. elongatum enabled us to investigate the organization and expression profiling of Tritipyrum WRKY genes across the entire genome.

**Results:**

In this study, 346 WRKY genes, from TtWRKY1 to TtWRKY346, were identified in Tritipyrum. The phylogenetic analysis grouped these genes into three subfamilies (I-III), and members of the same subfamilies shared a conserved motif composition. The 346 TtWRKY genes were dispersed unevenly across 28 chromosomes, with 218 duplicates. Analysis of synteny suggests that the WRKY gene family may have a common ancestor. Expression profiles derived from transcriptome data and qPCR demonstrated that 54 TtWRKY genes exhibited relatively high levels of expression across various salt stresses and recovery treatments. Tel1E01T143800 (TtWRKY256) is extremely sensitive to salt stress and is on the same evolutionary branch as the salt-tolerant A. thaliana genes AtWRKY25 and AtWRKY33. From 'Y1805', the novel AtWRKY25 was cloned. The Pearson correlation analysis identified 181 genes that were positively correlated (R>0.9) with the expression of TtWRKY256, and these genes were mainly enriched in metabolic processes, cellular processes, response to stimulus, biological regulation, and regulation of biological. Subcellular localization and qRT-PCR analysis revealed that TtWRKY256 was located in the nucleus and was highly expressed in roots, stems, and leaves under salt stress.

**Discussion:**

The above results suggest that TtWRKY256 may be associated with salt stress tolerance in plants and may be a valuable alien gene for improving salt tolerance in wheat.

## Introduction

Salinization of the soil is one of the principal abiotic stressors, as salt inhibits plant growth and development and affects food productivity (Van Zelm et al., 2020). On recent years, food production in high-yielding fields has slowed, but the vast majority of low- and medium-yielding farms have significant space for development. How to efficiently utilize saline and other low- and medium-yielding areas to boost total food production is a crucial issue that must be addressed. Salt stress can result in basic strains like as osmotic stress and ion toxicity, as well as secondary effects including oxidative stress and nutritional stress (Yang and Guo, 2018). Multiple pressures have an impact on cell growth and metabolic activities, which in turn affects seed germination, seedling growth, and crop output (Munns and Tester, 2008). Plants have evolved complex mechanisms to deal with salt stress at the morphological-structural, physiological-metabolic, and molecular levels, including reduced leaf number and area, stomatal closure, accumulation of osmoregulatory substances, Na^+^ and Cl^-^ efflux and compartmentalization, scavenging of reactive oxygen species, and modifications in stress-responsive gene expression (Dai et al., 2018; Hou et al., 2018). The expression of stress-responsive genes determines the degree to which morphological structure and physiological metabolic levels improve, and transcription factors play a crucial role in regulating this expression (Liang et al., 2018).

WRKY are specific plant transcription factors whose members are involved in a wide range of biological processes, including morphogenesis, biotic and abiotic stresses, seed germination, and plant senescence (Chen et al., 2017). All members of the WRKY transcription factor family contain a WRKY domain (WD) consisting of 60 amino acid residues. The N-terminal part of the domain is the WRKYGQK sequence, which is associated with DNA binding activity, and the C-terminal part of the domain is the C_2_H_2_ (C-X_4-5_-C-X_22-23_-H-X_1_-H) or C_2_HC (C-X_7_-C-X_23_-H-X_1_-C) zinc finger structure, which is involved in protein interactions and auxiliary DNA binding (Eulgem et al., 2000; Jiang et al., 2017b). The W-box is the shortest sequence necessary for the binding of WRKY transcription factors to DNA. The W-box has highly conserved C/TTGACT/C amino acid residues, of which TGAC is the most conserved region (Ülker and Somssich, 2004). Mutations in any of the nucleotides will affect the ability to bind WRKY transcription factors, which are also involved in the WRKY-W-box binding reaction through phosphorylation reactions, and the phosphorylation process requires the involvement of Zn^2+^ (Maeo et al., 2001; Duan et al., 2007). WRKY transcription factors are classified into three classes according to the number of structural domains and structural domain differences. Class I contains two WRKYGQK sequences, while classes II and III each contain one. Class I and class II have the same zinc finger structure as C-X_4-5_-C-X_22-23_-H-X_1_-H, while class III has a zinc finger structure of C-X_7_-C-X_23_-H-X_1_-C. Based on the differences in amino acid sequences, class II can be further subdivided into five subclasses: IIa, IIb, IIc, IId, and IIe (Yang et al., 1999).

A large number of salt-responsive WRKY transcription factors have been identified from a variety of plants, with subfamily IIc WRKY transcription factors showing key roles (Chen et al., 2017). Salt-responsive WRKY transcription factors drive or repress target gene transcription by interacting with W-box elements in promoters, participating in ABA, ethylene, and SOS signaling pathways, and acting as intermediate factors in the interaction of different signaling pathways. In *Arabidopsis*, overexpression of *AtWRKY25* and *AtWRKY33* genes can enhance plant salt tolerance by regulating the SOS pathway (Jiang and Deyholos, 2009; Rajappa et al., 2020), while expression of *AtWRKY8* is up-regulated under salt stress and enhances plant resistance to salt stress by activating the *RD29A* gene (Chen et al., 2013); In cotton, *GhWRKY17* can be induced by drought, salt, H_2_O_2_ and ABA, and its constitutive expression in tobacco suppresses the transcriptional levels of ABA-inducible genes (including AREB, DREB, NCED, ERD, LEA) and blocks the ABA signalling pathway (Yan et al., 2014). In response to salt stress, plants usually produce ROS, which mediates the response to salt stress through the ROS signaling process. *Sorghum bicolor SbWRKY50* can negatively regulate the salt response by reducing the expression level of the *Arabidopsis* Na^+^/H^+^ reverse transporter protein gene *AtSOS1* (Song et al., 2020). *Fortunella crassifolia FcWRKY40*, on the other hand, can directly activate the expression of *FcSOS2*, a serine/threonine protein kinase gene in the SOS pathway, and indirectly regulate the expression of *FcSOS1* and *FcSOS3* genes to promote Na^+^ efflux and positively regulate the response to salt stress. In addition, *FcWRKY40* can in turn be induced by ABA to be expressed as a target of the ABA response element binding factor *FcABF2*, and *FcWRKY40* may be a key transcription factor for the formation of cross-talk between the SOS and ABA pathways (Dai et al., 2018). In *GhWRKY17* overexpressing tobacco, the levels of ROS accumulation, electrolyte leakage, and malondialdehyde accumulation were significantly increased, and the expression levels of ROS scavenging genes, CAT and SOD genes, as well as proline content and enzyme activities were significantly decreased, thus reducing the tolerance of tobacco to salt stress (Yan et al., 2014). In *DgWRKY5* overexpressing chrysanthemum, the expression of ROS scavenging genes (including POD, CAT and SOD) was up-regulated, which improved the resistance of chrysanthemum to salt stress (Liang et al., 2017).

*T. aestivum* is a moderately salt-tolerant crop, with higher salt tolerance than rice but lower than barley, and is one of the major cultivated crops in saline lands. *Th. elongatum* is a close relative of *T. aestivum* and can grow in salt concentrations similar to seawater. The octoploid *Tritipyrum* obtained by intergeneric crosses between *T. aestivum* (AABBDD) and *Th. elongatum* (EE) is an important germplasm resource for introducing *Th. elongatum* salt tolerance genes into *T. aestivum*. The genomes of *T. aestivum* and T*h. elongatum* have been completely sequenced, giving a solid foundation for structural and functional investigations of the relevant genes (Mayer et al., 2014; Wang et al., 2020). In this study, the genomic structure characteristics, chromosomal positions, gene duplication, and evolutionary divergence of the WRKY gene family in *Tritipyrum* were investigated. In addition, the expression profiles of 54 *TtWRKY* genes were examined in response to salt stress. Finally, *TtWRKY256* was cloned and its subcellular location and expression levels during salt stress and recovery were determined. These results will pave the way for suggestions on how to improve salt tolerance in plants. These results will pave the way for suggestions on how to improve plant salt tolerance.

## Material and methods

### Plant material

*Tritipyrum* is a synthetic octoploid, which contains A, B, and D genomes from *Triticum aestivum* and a set of E genomes from *Thinopyrum elongatum*. Salt-tolerant ‘Y1805’ is a stable *Tritipyrum* octoploid from a wide cross between *T. aestivum* and *Th. elongatum*. The *Tritipyrum* protein and nucleic acid sequences used for identifying the WRKY genes in this study were obtained from *T. aestivum* genome database (http://plants.ensembl.org/Triticum_aestivum/Info/Index) and the *Th. elongatum* genome database (https://ngdc.cncb.ac.cn/gwh/Assembly/965/show). The genome sequences of *Arabidopsis thaliana*, *Hordeum vulgare*, *Oryza sativa*, *Zea mays*, and *Thinopyrum intermedium* were located in the Plant Genomics portal Phytozome13 (https://phytozome-next.jgi.doe.gov/). The *Secale cereal* genome sequences were obtained from the China National GeneBank DataBase (https://ngdc.cncb.ac.cn/gwh/Assembly/12832/show). Publicly transcriptome available datasets were analyzed in this study. This data can be found here: [https://dataview.ncbi.nlm.nih.gov/object/PRJNA769794?reviewer=7v7it5jpc4odu8mlni9frga27g/accessionnumber:PRJNA769794].

### Genomic *in situ* hybridization

Seeds were germinated at 25°C on moist filter paper in Petri dishes, then kept at 4°C for about 24 hours before being transferred to 25°C. Roots 1 to 2 cm in length were cut and treated in ice water for approximately 24 h before fixation in Carnoy’s solution. After fixation, the root tips were stained with carbol fuchsin, and their mitotic chromosomes were observed under a microscope. When the plants reached the flag leaf stage, spikes were sampled and anthers at metaphase I (MI) of meiosis were fixed in Carnoy’s solution, dissociated in 1 M HCl at 60°C for 6 to 8 min, and homogenized in 1% acetocarmine. *Th. elongatum* DNA was labeled with fluorescein-12-dUTP by the nick translation method and used as probes. Sheared genomic DNA from Chinese Spring (AABBDD, 2n = 42) was used as blocking DNA. In the Vectashield mounting medium (Vector Laboratories, USA), the slides were counterstained with propidium iodide (PI, 0.25 mg/mL).

### Identification of the WRKY genes in *Thinopyrum*

From the Pfam database (http://www.pfam.sanger.ac.uk/), the consensus protein sequences (PF03106) of the WRKY hidden Markov model (HMM) were downloaded. Additionally, a search file library was created from 68 reported AtWRKY sequences (Eulgem et al., 2000) that were acquired from the UniProt database (www.uniprot.org). With the published sequences of the WRKY proteins from *A. thaliana* and their WRKY domain as query sequences, we utilised the Basic Local Alignment Search Tool algorithm software (BLASTP) to look for *Thinopyrum* WRKY (*TtWRKY*) proteins. After culling duplicates, we used the Pfam database and the SMART tool (http://smart.embl-heidelberg.de/) to verify the validity of the remaining candidate sequences (Letunic et al., 2012). ExPASy (http://web.expasy.org/protparam/) was used to generate the genes’ physical and chemical properties.

### Phylogenetic analyses and conserved motif determination

The UniProt database was used to obtain *A. thaliana* WRKY protein sequences for phylogenetic trees. The Clustalx2.0 tool was used to align several amino acid sequences from discovered WRKY genes. The phylogenetic trees comparing *Tritipyrum* and *A. thaliana* were built using the NJ method, with the Poisson model and 1000 bootstrap replications as the particular parameters. The MEME online tool (http://meme.nbcr.net/meme/intro.html) was used to determine the conserved motifs in *TtWRKY* proteins, with the parameters set to the optimum mode width of 6 to 200 and the maximum number of motifs of 10. The phylogenetic tree was shown, edited, and coloured using FigTree software and iTOL (http://itol.embl.de/).

### Chromosomal distribution and gene duplication of the *TtWRKY* genes

The method of mapping *TtWRKY* genes to the chromosomes of *Tritipyrum* was performed according to Liu et al. Analysis of the *TtWRKY* gene replication events was conducted using multiple collinear scanning toolkits (MCScanX) using default parameters. All-vs-all protein sequence comparisons necessary for MCScanX were performed using DIAMOND v0.8.25 (–max-target-seqs 5 –evalue 0.00001) (Buchfink et al., 2015).

### Plant growth conditions and stress treatments

In a growth chamber (relative humidity of 75% and a 20/15°C light-dark photocycle), the seeds of ‘Y1805’ were germinated. The seedlings were seeded on a floater board in 1/2 Hoagland’s solution with a 16/8 h light/dark cycle, 400 μmol m^−2^s^−1^ irradiance, and the same temperature and relative humidity as the germination chamber. Every three days, the culture solution was renewed. On the fourteenth day (two-leaf stage), salt stress treatments were initiated (1/2 Hoagland’s solution supplemented with 250 mM NaCl). 5 hours after exposure to salt stress, the first root, stem, and leaf samples of uniform size were collected from *T. aestivum*. After 24 hours of salt stress, the materials were recovered (in 1/2 Hoagland’s solution without NaCl). The second sample was taken 1 hour after recovery. CK1 and CK2 were employed as parallel controls, both consisting of normal (1/2 Hoagland’s solution without NaCl) grown materials. All tissue samples were immediately frozen in liquid nitrogen and preserved at -80°C for qPCR, as well as gene cloning. Three biological replications were utilised, with at least ten seedlings per replicate being combined.

### Expression analysis and qPCR validation of WRKY genes under salt stresses and recovery

These RNA-Seq data sets were utilised for the salt stress and recovery expression investigation of *Tritipyrum* WRKY genes. Using FeatureCounts v1.5.1, reads corresponding to WRKY genes were tallied (Liao et al., 2014). Calculated read abundance as transcripts per kilobase million (TPM). The R Circlize package was used to log transform and visualise the results (https://cran.r-project.org/web/packages/circlize.html). GO annotation assignment was utilized to achieve functional gene annotation by mapping GO terms utilizing GO (http://www.geneontology.org/), and KEGG (http://www.genome.jp/kegg/) databases in the BLAST2GO tool (Biobam Bioinformatics S.L., Valencia, Spain, http://www.blast2go.com/b2ghome/about-blast2go) with an E-value threshold of 10^-6^. Primer 5.0 was used to design the primer sequences (**Table S2**). As an internal control, we utilized the *Actin* gene, which was consistently expressed at each growth stage in nearly all tissues. The *ACTIN* gene was utilized to calibrate the detection of three technical repeats of the three biological repeats, and technique 2^−ΔΔCt^ was employed to assess the expression. RNA reverse transcription, WRKY gene amplification, and plasmid construction

Utilizing a PrimeScript™ RT reagent Kit (Takara), RNA was reverse-transcribed into cDNA. The complete coding sequence of *TtWRKY256* was amplified from ‘Y1805’ cDNA using *Bsa* I restriction site-containing primers at the 5’ and 3’ ends of the amplified fragment. The amplification primers are presented in **Table S2**. Following the manufacturer’s instructions, the amplified fragment was digested with *Sac*/*Spe* I and *BamH* I/*Kpn* I and then inserted into the pBI121 vector using T4-DNA ligase (Takara). This vector has been altered to include the gene for green fluorescence protein (GFP). The inserted sequence was driven by the 35S promoter of CaMV.

### Sequence alignments and phylogenetic analysis of *TtWRKY256* gene

DNAMAN performed multiple alignments of the WRKY gene sequences. From Phytozome (https://phytozome.jgi.doe.gov/pz/portal.html) and Ensembl (http://plants.ensembl.org/Triticumaestivum/Info/Index) servers, 21 DNA sequences encoding for WRKYs were retrieved. With MEGA X and the maximum likelihood method and 1000 bootstrap replications, a phylogenetic tree was constructed (Kumar et al., 2018).

### Subcellular localization of *TtWRKY256*

The recombinant *TtWRKY256*-GFP and the vector pBI121-GFP as a negative control were infiltrated into tobacco epidermal cells *via Agrobacterium tumefaciens*-mediated transformation. After incubation for 24 h, the green fluorescent protein (GFP) fluorescence signal was observed using a confocal microscope (FV1000 Olympus Corp., Tokyo, Japan).

### Statistical analysis

SPSS software was used to conduct an analysis of variance (ANOVA) on the study’s data (IBM Corporation). At a significance level of 0.05, mean values were compared using Fisher’s least significant difference (LSD) test. The histograms were created with the Origin 8.0 program (OriginLab Corporation, Northampton, Massachusetts, USA).

## Results

### Identification of the *TtWRKY* genes in *Tritipyrum*

**Figure 1:**
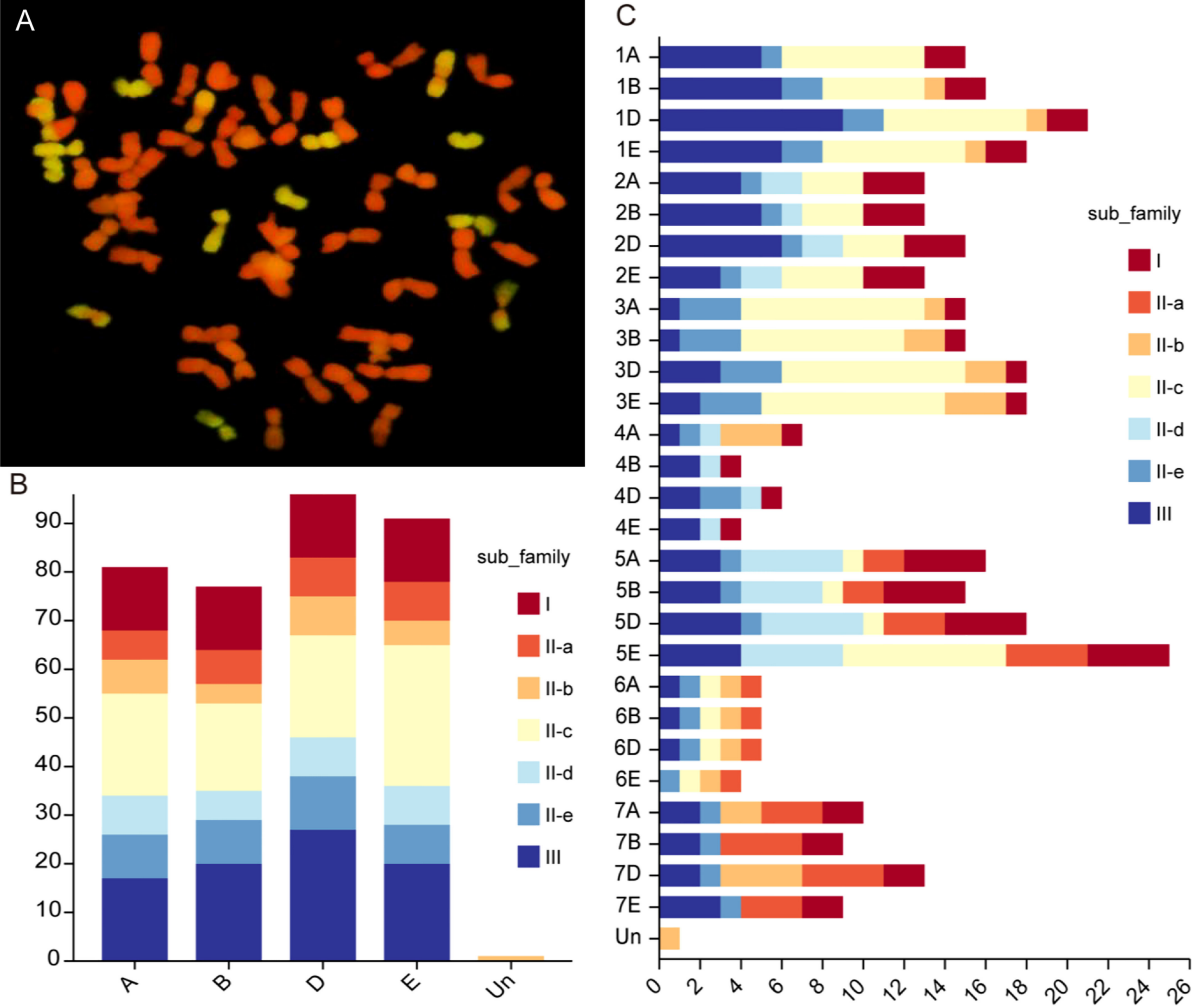
Chromosome configuration and distribution of WRKY genes in *Tritipyrum.* **(A)** Chromosome configuration of *Tritipyrum* ‘Y1805’; **(B)** Subgenomes distribution of WRKY genes in *Tritipyrum*; **(C)** Chromosomal distribution of WRKY genes in *Tritipyrum*.

Cytogenetic analysis revealed that *Tritipyrum* ‘Y1805’ had 56 chromosomes, 42 of which were from *T. aestivum* (red) and 14 from *Th. elongatum* (green) (**Figure 1A**). After eliminating the duplicated sequences, 346 WRKY genes were retrieved from the *Tritipyrum* genome using the hidden Markov model (HMM) and BLASTp approaches in this study. WRKY genes were renamed based on where they were location on *Tritipyrum* chromosomes (**Supplementary Table S1**). The results showed that the 346 WRKY genes identified in *Tritipyrum* were mainly distributed in D subgenomes and five homologous groups (**Figures 1B, C**). The predicted WRKY proteins of *Tritipyrum* varied greatly in length and molecular weight (MV). The *Tritipyrum* WRKY genes encoded proteins ranging from 135 (TraesCS1A02G348600.1 and TraesCS1D02G351600.1) to 1482 (TraesCS5A02G344100.1) amino acids (aa) in length and from 14.93 (TraesCS1D02G351600.1) to 165.34 (FtPinG0000377600.01) kDa in MV. The isoelectric points (PIs) of the proteins ranged from 4.64 (TraesCS3D02G289500.1) to 10.86 (TraesCS5D02G070700.1) (**Supplementary Table S1**).

### Phylogenetic analysis, and motif composition of the *TtWRKY* gene

**Figure 2:**
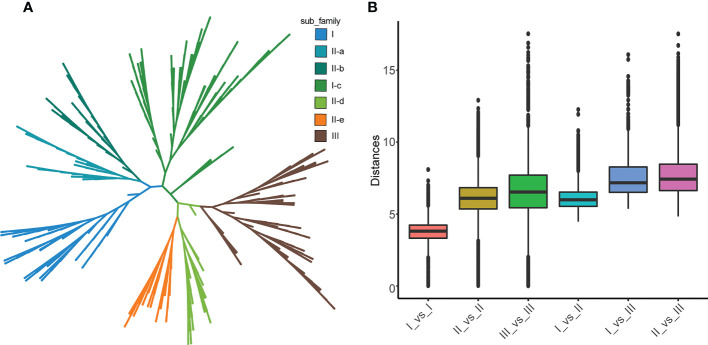
phylogenetic relationships and distance among the WRKY proteins from *Tritipyrum* and *A thaliana.* **(A)** Phylogenetic relationships among 414 WRKY proteins from *Tritipyrum* and *A thaliana*; **(B)** genetic distance among the different clades of WRKY genes. The box plot shows the median (black line), interquartile range (box), and maximum and minimum scores (whiskers) of each data set.

According to the phylogenetic tree and motif composition, these gene families were divided into seven subfamilies (**Figure 2A**; **Figure S1**). To investigate the evolutionary relationships and classify the WRKY family in *Tritipyrum*, a phylogenetic tree was constructed using 414 potential WRKY structural domains identified in *A. thaliana* and *Tritipyrum* (**Figure 2A**; **Figure S1**). According to the *AtWRKY* classification and primary amino acid sequence characteristics in the model organism *A. thaliana*, the WRKY family members in *Tritipyrum* could be divided into three major groups (Group I-III). Among them, 52 *TtWRKY* containing 2 WDs and 1 C2H2-type zinc finger structure belong to Group I; Group II contains 210 members with 1 WD and C2H2-type zinc finger structure in each sequence, and the family can be further divided into 5 subfamilies IIa, IIb, IIc, IId, and IIe, containing 29, 25, 89, 30, and 37 members; 84 *TtWRKY* contained 1 WD and C2HC-type zinc finger structure and belonged to Group III. In this study, Group II was the largest WRKY transcription factor family in *Tritipyrum*, accounting for 60.7% of the total, and subfamilies IIa and IIc and IId and IIe clustered to one branch, respectively, which was consistent with previous studies in *T. aestivum*. This is consistent with the results of previous studies in *T. aestivum*. The box plot indicates that the genetic distance I_vs_II is smaller than the genetic distance I_vs_III and II_vs_III, and that the genetic distance between Is is the smallest, indicating that Is and IIs are more closely related and that the relationship between Is is the closest (**Figure 2B**). In addition, the genetic distances of IIs and IIIs were comparable, but the range of IIs was the smallest, indicating that IIs had less sequence divergence than IIIs (**Figure 2B**).

A total of 10 different motifs, named motif 1-10 (**Figure 2A**; **Figure S1**), were identified using the MEME online program. Motif 1 and motif 6 both contain the characteristic sequence WRKYGQK of WRKY proteins, and almost all identified WRKY proteins contain at least one of these motifs. Motif anlysis indicates that WRKY proteins of the same family or subfamily in *Tritipyrum* have similar motif composition. For example, motif 9 is unique to Group I, while motif 8 is unique to Group IIa and Group IIb. The motifs specific to a family may be involved in plant-specific biological processes. Thus, each family or subfamily of WRKY genes may be associated with a specific biological process. Motif distribution patterns of members of subfamilies IIa and IIb are the same, indicating that their functions are similar. These two families were also clustered onto the same branch in the phylogenetic tree, and the same phenomenon was observed for the IId and IIe subfamilies. These results further validate the classification of WRKY in *Tritipyrum* and its phylogenetic relationships.

### Chromosomal distribution, gene duplication and synteny analysis of the *TtWRKY* gene

**Figure 3:**
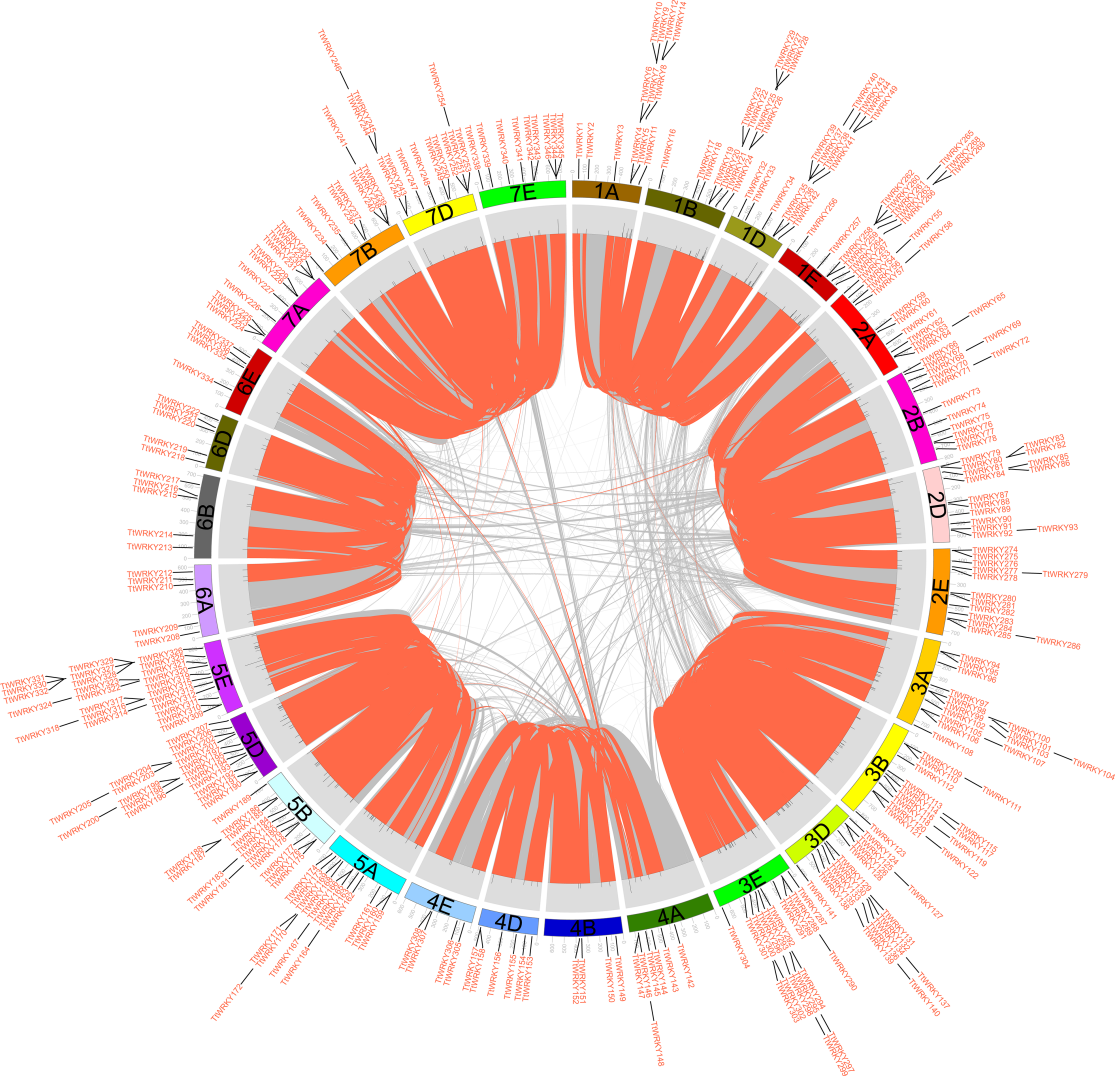
distribution, duplication and synteny analysis of WRKY genes in *Tritipyrum.* Collinear correlations of WRKY in *Tritipyrum* genomes are displayed by Circos. *Tritipyrum* chromosomes are colored according to the inferred ancestral chromosomes following an established convention. In the center, the relative map position of 345 WRKY genes is shown on each of the 28 *Tritipyrum* chromosomes.

Of the 346 *TtWRKY* genes, 345 were localized to 28 chromosomes, and only *TtWRKY255* was not shown in the chromosome localization map due to anchoring on scaffolds (**Supplementary Table S1**; **Figure 3**). Most of the *TtWRKY* genes were distributed on chromosomes 5E (25, 7.22%) and 1D (21, 6.06%), while chromosomes 4B, 4E and 6E contained only four *TtWRKY* genes (**Supplementary Table S1**; **Figure 1**, **3**). Based on where they were on the chromosome, most of the *TtWRKY* genes were at the ends, and only a few were close to the near-central region (**Figure 3**) The above results indicated that the localization of WRKY genes on chromosomes was non-random and unevenly distributed. In this study, a total of 405 *TtWRKY* gene duplication pairs were identified, including 218 genes (**Figure 3**). This suggests that some genes contain more than one duplicate gene, probably due to multiple replication throughout *T. aestivum* genome. The tandem duplicated gene pairs were mainly distributed in the first, third, and fifth homologous groups, with the vast majority of homologous genes distributed on the same homologous groups and only a few in the fourth, fifth, and seventh homologous groups, which is consistent with the natural translocations generated during the formation and evolution of common *T. aestivum*.

### Evolutionary analysis of the WRKY Families in several different species

**Figure 4:**
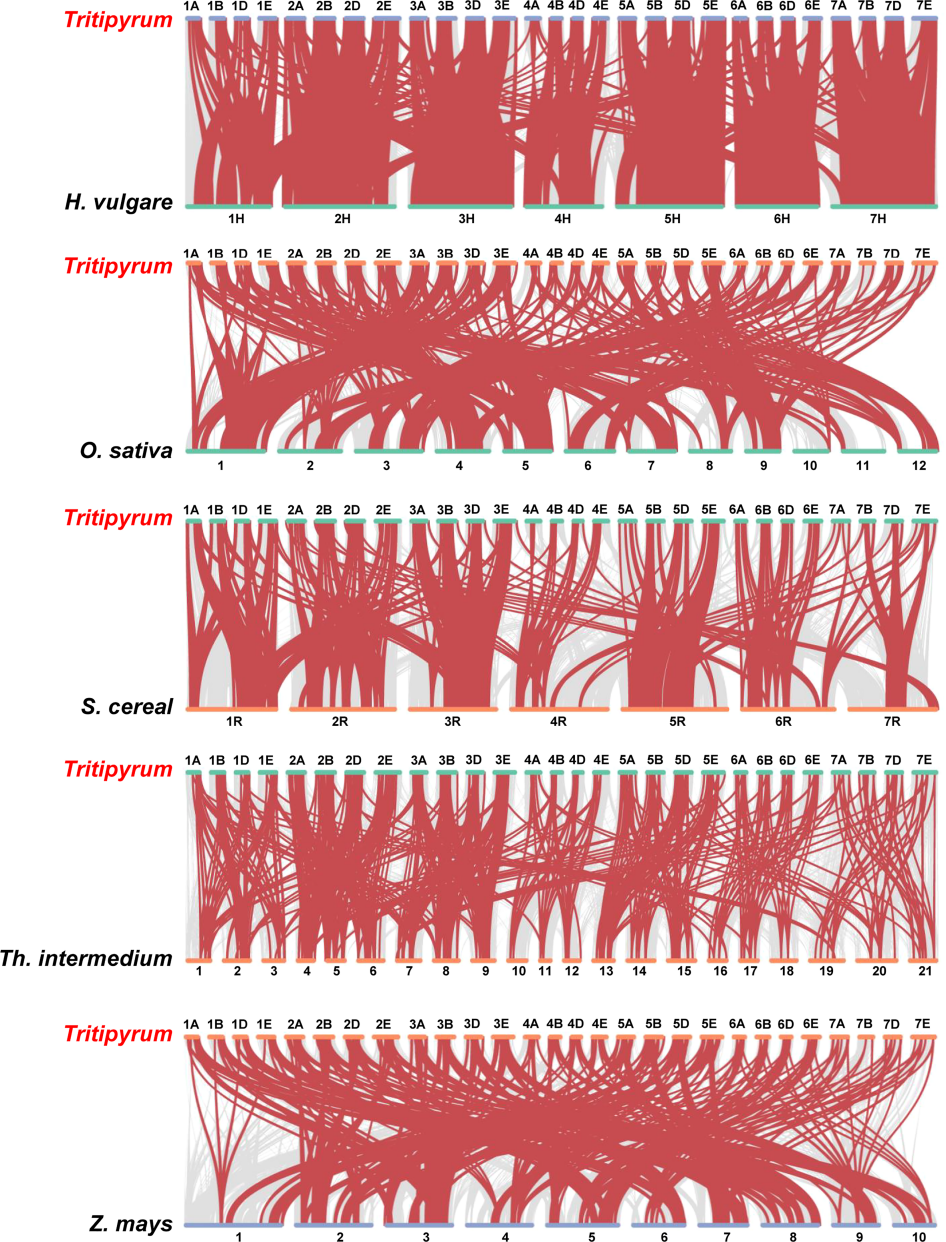
Synteny analyses between *Tritipyrum* and five representative plant species. Gray lines in the background indicate collinear blocks within *Tritipyrum* and other plant genomes, while red lines highlight syntenic WRKY gene pairs.

To further deduce the evolutionary ties between members of the WRKY gene family in *Tritipyrum*, *H. vulgare*, *O. sativa*, *S. cereal*, *Th. intermedium*, and *Z. mays*, the syntenic relationships between the six species were studied. Five distinct classes of syntenic linkages were identified (**Figure 4**). 255 *TtWRKY* genes shared syntenic connections with those of *Th. intermedium*, followed by *Z. mays* (251), *O. sativa* (262), *H. vulgaris* (218), and *S. cereal* (185). (**Figure 4**). Intriguingly, collinear pairs were discovered between *Tritipyrum* and the other five species, suggesting that these orthologous pairs may have existed prior to the original separation. In addition, certain WRKY collinear gene pairs found between *Tritipyrum* and *H. vulgare* were anchored to the highly conserved syntenic blocks, which encompass over 500 collinear sites. Similar trends were also identified between *Tritipyrum* and *S. cereal*, which may be attributable to the evolutionary connection between *Tritipyrum* and the other five plant species. Significantly, several *TtWRKY* genes were discovered to be connected with at least three syntenic gene pairs, indicating that these genes may have played a crucial role in the evolution of the WRKY gene family. These results revealed that the *TtWRKY* gene family was highly conserved and that the *TtWRKY* genes were more closely related to those of *H. vulgare* than to those of *Z. mays*. The *TtWRKY* genes in several plants may have developed from a common ancestor.

### Expression of *TtWRKY* genes under salt stresses and recovery

**Figure 5:**
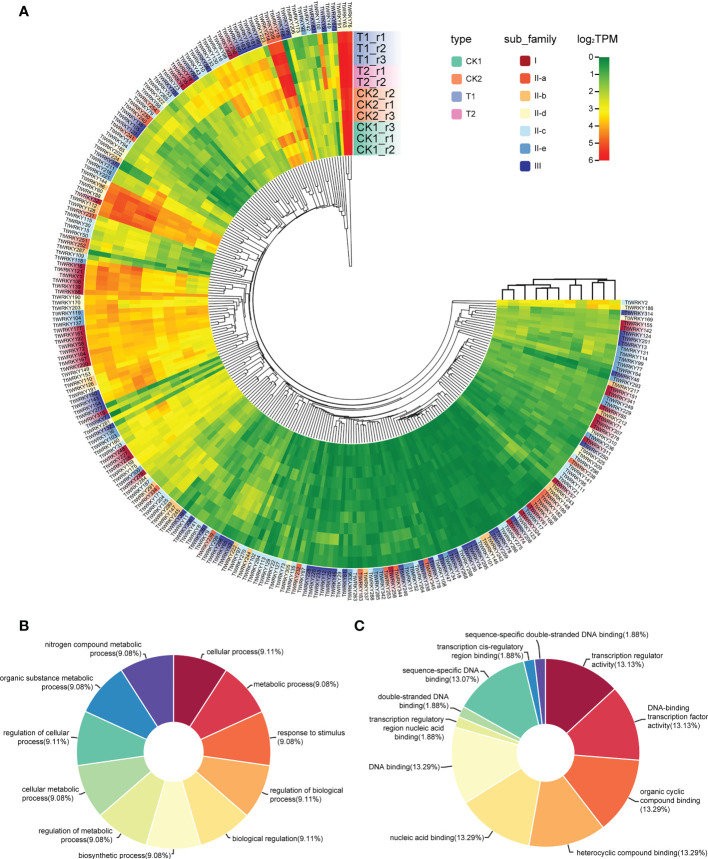
Expression patterns of *TtWRKY* genes under salt stresses and recovery treatments. **(A)** Hierachical clustering of expression profiles of 249 *TtWRKY* genes were expressed in 11 samples including salt stress and recovery treatment. **(B, C)** the BP **(B)** and MF **(C)** analysis of 249 expression genes.

To confirm whether the expression of *TtWRKY* genes was influenced by different salt stresses and recovery treatments, the transcriptional levels of all 346 *TtWRKY* genes under different salt stresses and recovery treatments were investigated. Among the 346 *TtWRKY* genes, 249 *TtWRKY* genes were expressed in all 11 samples tested, and 107 genes showed constitutive expression (TPM>1 in all samples). These 249 *TtWRKY* genes were mainly from Group IIc and Group III subfamilies, and the clustering analysis showed that the corresponding WRKY genes were not significantly correlated with subfamily types for salt stress and recovery treatments (**Figure 5A**). 87 *TtWRKY* genes were not expressed in all detected samples, which may be pseudogenes or not a response to salt stress and recovery treatment. GO annotation of 249 expressed genes showed that the biological process and molecular function of these genes was cellular process, metabolic process, response to stimulus, regulation of biological process, biological regulation, biosynthetic process, regulation of metabolic process, cellular metabolic process and transcription regulator activity, DNA-binding transcription factor activity, organic cyclic compound binding, heterocyclic compound binding, nucleic acid binding, DNA binding (**Figures 5B, C**).

**Figure 6:**
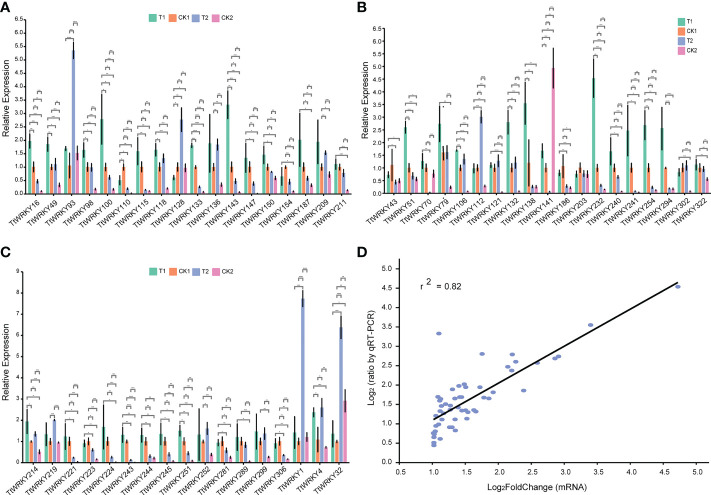
**(A–C)** Expression analysis of 54 WRKY genes in eleven samples by qPCR. Data were normalized to β-actin gene and vertical bars indicate standard deviation. **(D)** The relationships between qPCR and transcriptional of 54 up-regulated expression genes. Values are the log_2_ratio (salt stress or recovery treatment/CK treatment) for genes. The determine coefficient (r^2^) is indicated in the figure. All qPCR reactions were performed in three biological replicates. Asterisk, double and triple asterisks indicate significant differences (p < 0.05, 0.01 and 0.001, respectively) between groups.

To further verify the accuracy of transcriptome data, 54 *TtWRKY* members, whose mRNA levels were relatively high (log_2_FoldChange>1) across different salt stresses and recovery treatments, were carefully selected from 249 *Tritipyrum* WRKY genes. We designed specific primers for the fifty-four genes (**Supplementary Table S2**). Quantitative real-time PCR (qPCR) experiments were further performed to analyze their expression patterns in response to different salt stress and recovery treatments. Quantitative variations of the selected genes between qPCR and transcriptome were roughly similar (**Figures 6A–C**); gene expression trends were significantly similar (r^2^ = 0.82) with those from the transcriptome data, indicating that our transcriptome results were reliable (**Figure 6D**).

### *TtWRKY256* cloning and sequence analysis

**Figure 7:**
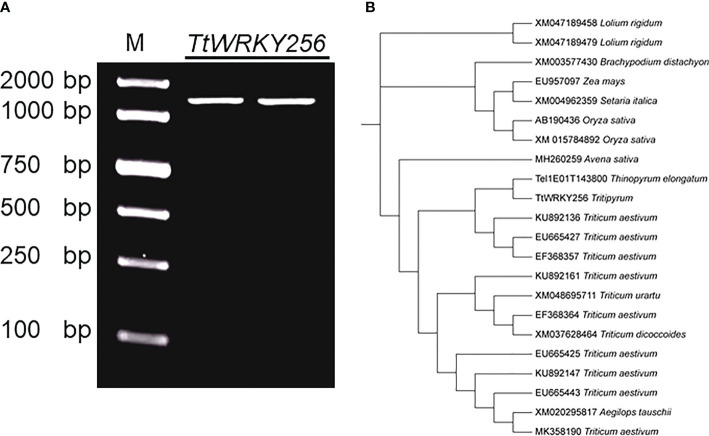
Clone and Phylogenetic relationships of *TtWRKY256.* **(A)** Amplification products using ‘Y1805’ cDNA; **(B)** Phylogenetic tree of WRKY nucleic acid sequences in various plant species. The Maximum Likelihood (ML) tree was generated using MEGA X with 1000 bootstrap replicates.

Earlier studies showed that overexpression of *AtWRKY25* (AT5G07100) and *AtWRKY33* (AT2G38470) genes in *A. thaliana* enhances plant salt tolerance by regulating the SOS pathway. *TtWRKY256* (Tel1E01T143800) gene of the *Tritipyrum* WRKY gene family and AT5G07100 and AT2G38470 are located in the same evolutionary tree branch (**Figure S1**) and are up-regulated in different salt stresses and recovery treatments (**Figure 5**). Using Tel1E01T143800-specific primers, a 1422 bp cDNA fragment corresponding to Tel1E01T143800was amplified and cloned from *Tritipyrum* ‘Y1805’ by PCR (**Figure S2**) and named *TtWRKY256*. The *TtWRKY256* sequence had 99.41% identity to Tel1E01T143800, with only 7 bp nucleotides changes between them (**Figure 7A**). Therefore, *TtWRKY256* was similar to Tel1E01T143800 according to their cDNA sequences. A phylogenetic tree based on nucleic acid sequences of different species showed that *TtWRKY256* displayed maximum homology with *Th. elongatum* (Tel1E01T143800, **Figure 7B**). The genetic distance of *TtWRKY256* with *T.aestivum* was closer than *T.urartu*, *T.dicoccoides* and *A.tauschii*. The *Triticum* crops and *A.sativa* were clustered together, with *O.sativa*, *Z.mays*, *S.italica*, *B.distachyon* and *L.rigidum* being more distantly related, which is consistent with the distant affinities among the species (**Figure 7B**).

### *TtWRKY256* expression correlation analysis

**Figure 8:**
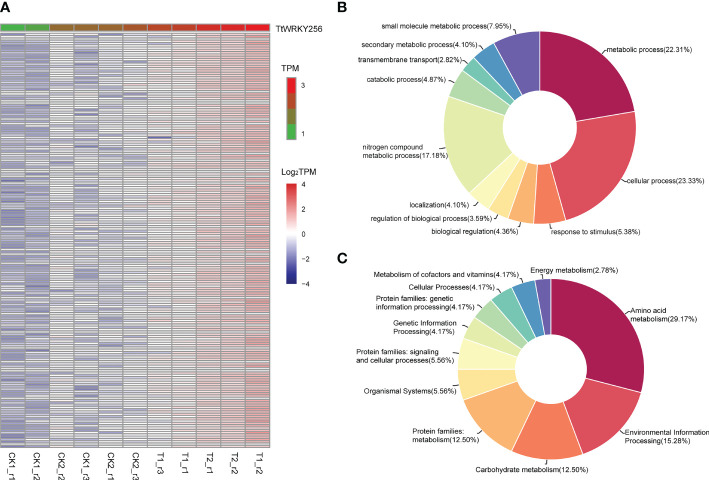
Expression correlation analysis of *TtWRKY256.* **(A)** One hundred eighty-one genes positively related (R > 0.9) with *TtWRKY256* expression; **(B, C)** the BP **(B)** and KEGG **(C)** analysis of 181 positively related to *TtWRKY256* expression genes.

To explore the biological processes associated with *TtWRKY256* expression in salt stress and recovery treatments, a Pearson correlation analysis was performed on *TtWRKY256* and other genes in transcriptome data. The results showed that 181 genes (R > 0.9) were positively correlated with the expression of *TtWRKY256* (**Figure 8A**). Biological process (BP) and KEGG analyses were performed on these 181 genes by Blast2GO software and the KEGG website (https://www.genome.jp/kegg/). The results are listed in **Figures 8B, C**, wherein 5 BPs for 173 genes were observed, including metabolic process, cellular process, response to stimulus, biological regulation, and regulation of biological process. As for KEGG analysis, amino acid metabolism, environmental information processing, carbohydrate metabolism, protein families: metabolism, organismal systems, and protein families: signaling and cellular processes were included. All the above results indicated that *TtWRKY256* could be associated with abiotic stress tolerance in plants.

### Expression patterns of *TtWRKY256* and subcellular localization

**Figure 9:**
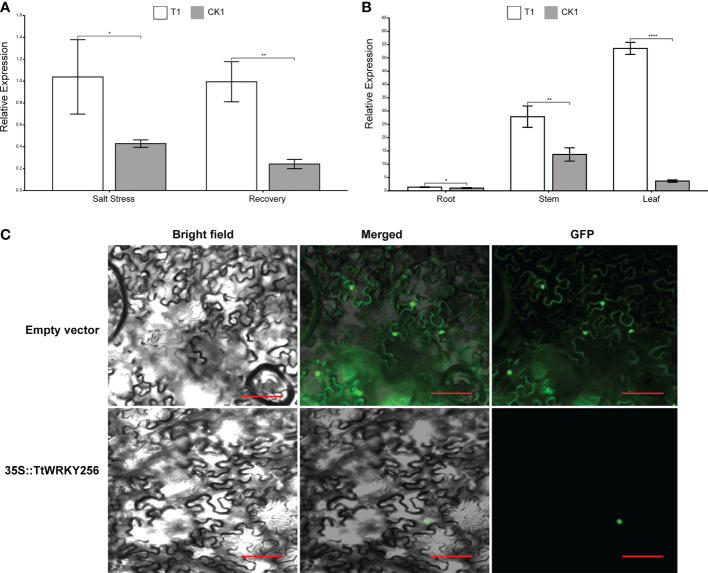
Expression and Subcellular localization of *TtWRKY256.* **(A)** Relative expression levels of *TtWRKY256* in roots under salt stress and recovery conditions; **(B)** Relative expression levels of *TtWRKY256* in roots, stems, and leaves under salt stress; **(C)** Subcellular localization of the TtWRKY256 protein in tobacco protoplasts, Scale bar = 20 mm. Asterisk, double and triple asterisks indicate significant differences (p < 0.05, 0.01 and 0.001, respectively) between groups.

To investigate the spatial and temporal expression pattern of *TtWRKY256*, qPCR analysis was used to examine the expression levels of *TtWRKY256* in roots under salt stress and recovery conditions and in diverse tissues. The *TtWRKY256* expression level was significantly (4.1-fold) higher than the control under salt stress and recovery in the roots (**Figure 9A**). These results were consistent with the above transcriptome data. In addition, the relative expression level of the *TtWRKY256* gene was the highest under salt stress in the leaves of Y1805, followed by stems, and then roots (**Figure 9B**). In short, all of the above results showed that the ‘Y1805’-specific *TtWRKY256* gene was highly and sensitively expressed in the whole plant when salt stress was high. To determine the subcellular localization of *TtWRKY256*, a fusion protein transiently expressing 35S-*TtWRKY256*-GFP in tobacco epidermal cells was produced. It was found that the fluorescence emitted by the fusion protein was localized to the nucleus (**Figure 9C**). The results showed that *TtWRKY256* might contribute to transcription regulation or the protection of heredity substances and cellular components.

## Discussion

In numerous plant species, the evolution, function, abiotic stress response, and other features of WRKY, one of the largest families of plant transcription factors, have been studied. In the present work, 346 members of the WRKY gene family were discovered in *Tritipyrum*. The 346 WRKY genes discovered in *Tritipyrum* can be categorized into three distinct groups. The proportion of WRKY genes represented by each group varied; group II contained the highest proportion of WRKY genes. Meanwhile, the genetic distances of IIs and IIIs were similar, which might be due to the group IIb originated from group III by the duplication of WRKY genes (Brand et al., 2013); the evolutionary patterns of this WRKY transcription factor have also been validated in wild rice (Jiang et al., 2017a). MEME analysis of WRKY protein sequences uncovered distinct group specificities. Group IIc WRKY proteins, for instance, are highly conserved and contain few additional motifs. Duplication of genes is essential for species evolution, genome amplification, and gene family evolution (Lynch and Conery, 2000). Whole genome duplication, tandem duplication, and segmental duplication are the three primary kinds of gene duplication (Yu et al., 2005). Genes that are duplicated may have distinct expressions. It seems that some evolutionary events such as duplication and polyploidy in plant have been extended the gene family members (Rezaee et al., 2020; Faraji et al., 2021). On the other hand, some changes in gene structure including point mutations in coding DNA sequence regions and regulatory site of duplicated members have affected the function of new members (Faraji et al., 2020; Heidari et al., 2021). The synteny of the WRKY genomes of six species, including *Tritipyrum*, *H. vulgare*, *O. sativa*, *S. cereal*, *Th. intermedium*, and *Z. mays*, was studied. The most syntenic linkages were discovered between *Tritipyrum* and *Th. intermedium*. This data implies that these taxa share a tight evolutionary relationship, compatible with traditional Gramineae classifications. *Tritipyrum* and *S. cereal*, which belong to separate genera, were determined to have the fewest number of syntenic links. In the five sets of syntenic relationships, 1-17 WRKY genes were shared by at least three species, which may shed light on the evolution of WRKY genes across species.

Salinity is a significant environmental danger to crop production because the excessive concentration of salt in the soil has a devastating impact on plant performance by disrupting cellular metabolism. These negative consequences of increasing salinity are mostly caused by osmotic stress and the accumulation of harmful ions in plant cells. Salinity is a major environmental threat for crop production because high concentration of salt in the soil severely affects plant performance by disturbing cellular metabolism. Such adverse effects of increased salinity occur mainly due to the osmotic stress and continuous accumulation of toxic ions within the plant cells (Munns and Tester, 2008). Genes encoding TFs are differentially expressed in plants as part of their complex stress response systems. Several transcription factors, including bHLH, WRKY, MYC, NAC, MYB, and ERF/AP2, have been linked to salt tolerance pathways (Okushima et al., 2007; Golldack et al., 2011; Lan Thi Hoang et al., 2017; Meraj et al., 2020). Many WRKY genes have been identified in Arabidopsis, rice, maize, and wheat, respectively, in response to various adversity stresses. After overexpression of the *ZmWRKY106* gene, the drought tolerance of *A. thaliana* was improved. Under drought conditions (Wang et al., 2018). *AtWRKY53* can accelerate the metabolism of starch in the guard cells and reduce H_2_O_2_ levels, hence promoting stomatal movement (Sun and Yu, 2015). *TaWRKY44* has multiple abiotic stress tolerance in transgenic tobacco, including drought, salt, and osmotic stress (Wang et al., 2015). Twelve GmWRKY genes appear differentially expressed in soybean under salt stress (Song et al., 2016). Plant WRKY genes with generally high expression could activate transcription of downstream target genes and thus regulate plant growth and development (Xu et al., 2016). Tissue-specific expression of WRKY genes may affect the growth and developmental processes of target tissues/organs by regulating transcriptional processes (Tang et al., 2014). In this study, 249 *TtWRKY* genes were detected in *Tritipyrum* root tissues that significantly responded to the induction of salt stress, of which 107 *TtWRKY* genes had constitutive expression characteristics, and these *TtWRKY*s may be involved in the regulation of cellular basal life processes. Fifty-four of these *TtWRKY* genes were further selected to examine their role in salt stress response. The results of expression profiling and qPCR analysis showed that these 54 *TtWRKYs* may positively regulate salt stress response in *Tritipyrum* root tissues. Transgenic experiments will be used to learn more about the exact biological functions of these *TtWRKYs*, and their use in genetic engineering to improve crop stress resistance and other agronomic traits will also be looked into.

Similar to the previous investigation of *T. aestivum*, which identified 12 TaWRKY genes as candidate genes for salt stress response, the majority of *TtWRKY* genes responded to salinity stress (Ning et al., 2017). Overexpression of *AtWRKY33* has also been demonstrated to increase *A. thaliana* tolerance to salt stress (Bao et al., 2018). *AtWRKY33* is controlled not only by salt but also by oxidative stress (Jiang and Deyholos, 2009; Birkenbihl et al., 2012). In addition, genes regulated by *AtWRKY33* are associated with ROS detoxification processes, indicating that WRKY TFs are essential regulators for stress adaptation (Jiang and Deyholos, 2009). The *TtWRKY256* gene in the WRKY gene family of *Tritipyrum* and *AtWRKY33* in *A. thaliana* are located in the same evolutionary branch, and the *TtWRKY256* gene is up-regulated in different salt stresses and recovery treatments. As a result, *TtWRKY256* was selected for additional functional research in this experiment. Here, the relative expression level of the *TtWRKY256* gene was the highest in the leaves of *Tritipyrum* under salt stress, followed by stems, and then roots. It was found that the root system was damaged directly and seriously in a high salt solution. In addition, the *TtWRKY256* expression level in the whole plant was significantly higher than in the control under salt stress and recovery. These results were consistent with our transcriptome data and previous reports (Ning et al., 2017). Because of this, *TtWRKY256* was highly and sensitively expressed in the whole plant to help the plant deal with salt stress. GO and KEGG analysis of highly related genes of *TtWRKY256* demonstrated that the highly related genes are associated with in metabolic process, cellular process, response to stimulus, biological regulation, and regulation of biological process, which may contribute to the study of the salt tolerance mechanism of *TtWRKY256*.

Soil salinization is a growing problem and has become a major factor limiting seed germination, seedling growth and crop yield. *Th. elongatum* is a closely related species of *T. aestivum* that can grow in salt concentrations similar to those of seawater. *Tritipyrum*, obtained by intergeneric hybridization between *T. aestivum* and *Th. elongatum*, is an important bridge material for introducing salt tolerance genes of *Th. elongatum* into *T. aestivum* (Baker et al., 2020; McKenna et al., 2020). Improving salt tolerance in plants mainly induces the activation of stress-responsive genes, whose expression products are involved in the repair of various aspects of primary and secondary stresses induced by salt stress. In contrast to single functional genes, transcription factor can regulate a set of downstream target genes, which in turn regulate physiological and biochemical processes in response to salt stress (Jiang et al., 2017b; Lan Thi Hoang et al., 2017). In this study, bioinformatics such as phylogenetic analysis, motif analysis and correlation analysis were used to conduct a comprehensive analysis of the WRKY family in *Tritipyrum*. RNA-seq was used to look for WRKY transcription factors in *Tritipyrum* salt stress response as part of a transcriptomic analysis of the plant’s response to salt stress. The *TtWRKY256* gene was cloned and identified using the Arabidopsis salt stress response-related gene *AtWRKY33* as a reference gene, and the analysis of gene expression levels and subcellular localization of the *TtWRKY256* gene under salt stress and recovery were completed. The response of plants to salt stress is very complex, with multiple genes involved in the regulatory network and multiple pathways acting together. The functions of the *TtWRKY* transcription factors that were screened in this paper have not been transgenically characterized. It needs to be found out if these transcription factors interact with each other, if they interact with other proteins, and if they have downstream target genes. With the discovery and wide use of salt tolerance-related transcription factor candidate genes and the continuous improvement of the understanding of the salt tolerance mechanism involved in transcription factors, genetic engineering will make it easier to grow crops that can handle salt.

## Conclusions

In this study, a thorough examination of the WRKY gene family in *Tritipyrum* was conducted. 346 full-length WRKY genes were described and categorized further into three primary categories, with extremely similar motif compositions within the same groups and subgroups. Synteny analysis and phylogenetic comparison of WRKY genes from a variety of plant species yielded useful insights into the evolutionary properties of WRKY genes in *Tritipyrum*. Fifty-four *TtWRKY* genes play an important role in salt stress response in *Tritipyrum*, as evidenced by their expression patterns in different tissues and in response to salt stress and recovery treatments. In addition, *TtWRKY256* may be a potential target gene for enhancing wheat’s salt tolerance *via* biotechnology or molecular breeding. These data provide a great resource for gaining a deeper comprehension of the biological functions of particular WRKY genes in *Tritipyrum*.

## Data availability statement

The datasets presented in this study can be found in online repositories. The names of the repository/repositories and accession number(s) can be found in the article/**Supplementary Material**.

## Author contributions

KL planned and designed the research and analysed the data. KL and XL wrote the manuscript. FH, SC, and GZ studied gene expression by qPCR. FH identified the *Tritipyrum* WRKY gene family and analysed gene structure. YW and SZ studied chromosome distribution and gene duplication. YY analysed the evolutionary relationship of WRKY genes in several different species. MR supervised the research. YY and MR revised the manuscript. All authors read and approved the final manuscript. All authors contributed to the article and approved the submitted version.
