# Supplementary figures and images for: Genome-wide analysis of the Tritipyrum WRKY gene family and the response of TtWRKY256 in salt-tolerance
Source: Front Plant Sci. 2022 Dec 14;13:1042078. doi: 10.3389/fpls.2022.1042078 (PMC9795024; doi:10.3389/fpls.2022.1042078)

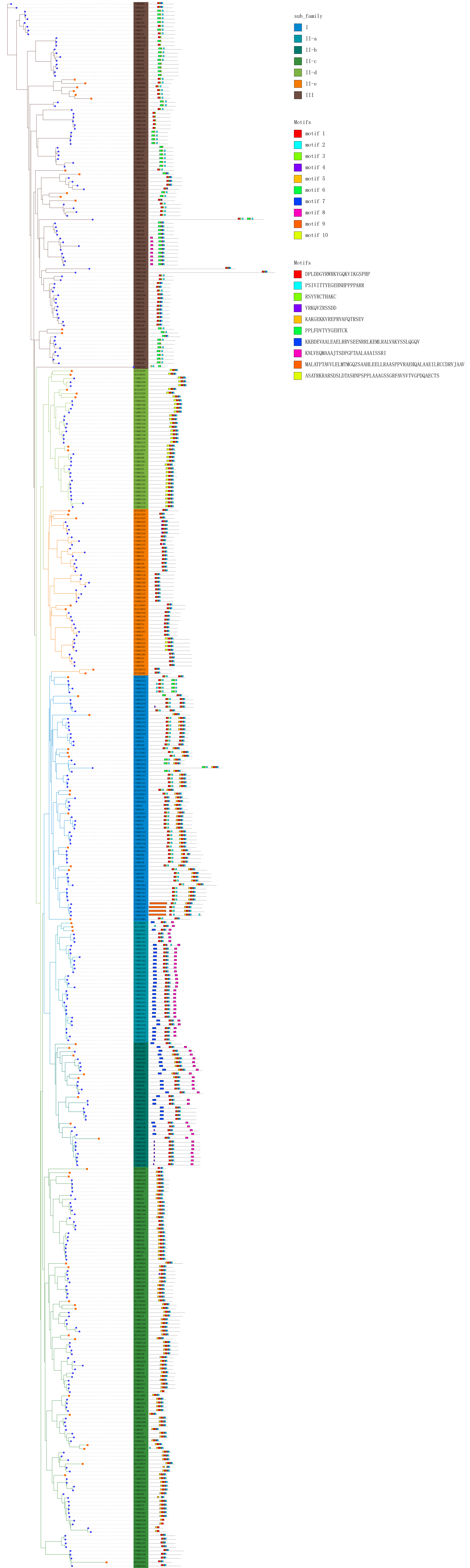

Supplement: Supplementary file 1 [file Image_1.pdf]

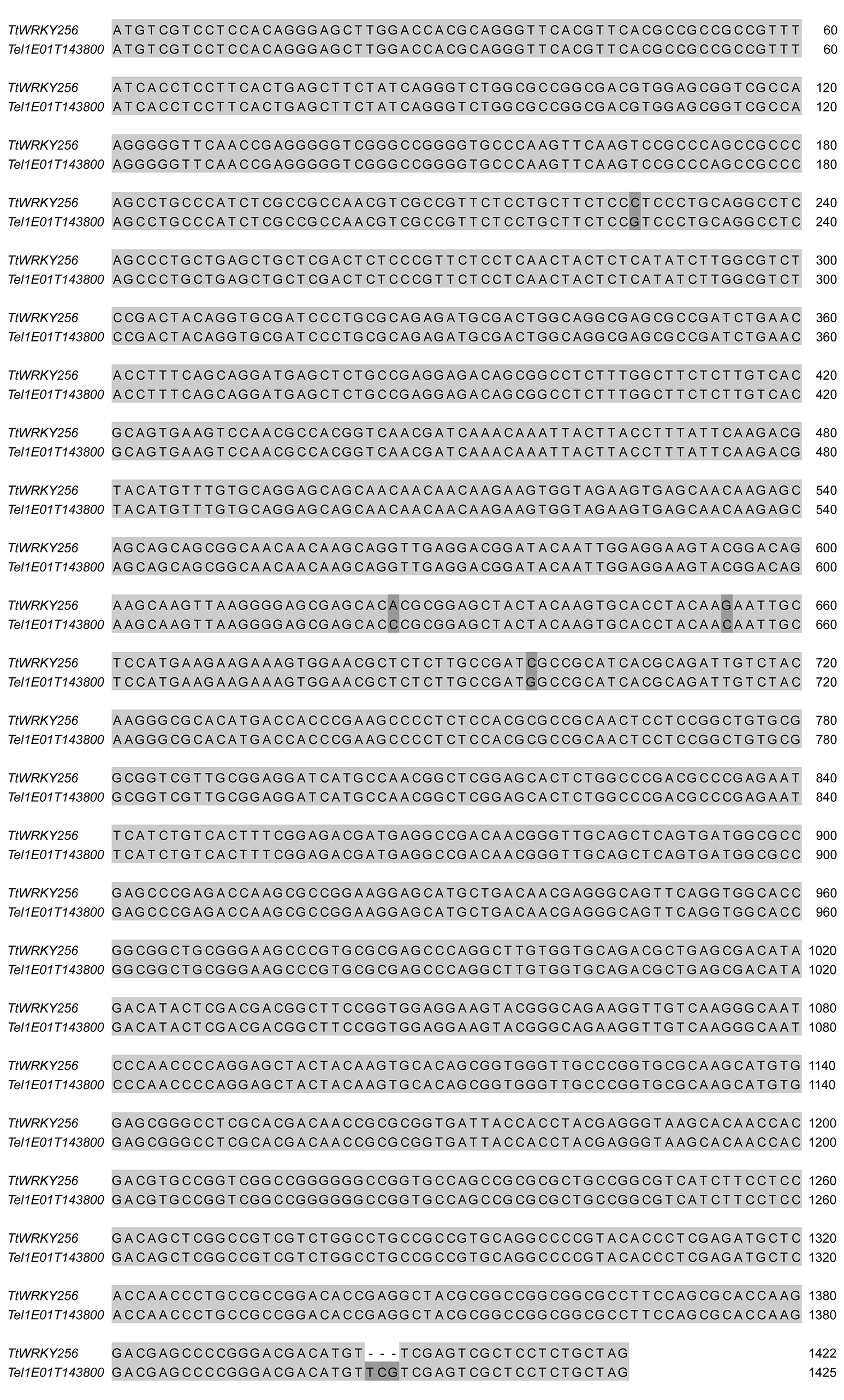

Supplement: Supplementary file 2 [file Image_2.tif]
